# Starvation during pregnancy impairs fetal oogenesis and folliculogenesis in offspring in the mouse

**DOI:** 10.1038/s41419-018-0492-2

**Published:** 2018-04-18

**Authors:** Jun-Jie Wang, Xiao-Wei Yu, Rui-Ying Wu, Xiao-Feng Sun, Shun-Feng Cheng, Wei Ge, Jing-Cai Liu, Ya-Peng Li, Jing Liu, Shu-Hua Zou, Massimo De Felici, Wei Shen

**Affiliations:** 1 Institute of Reproductive Sciences, College of Life Sciences, Qingdao Agricultural University, Qingdao, 266109 China; 2 Center for Reproductive Medicine, Qingdao Women’s and Children’s Hospital, Qingdao University, Qingdao, 266034 China; 3 Core Laboratories of Qingdao Agricultural University, Qingdao, 266109 China; 4 Department of Biomedicine and Prevention, University of Rome Tor Vergata, Rome, 00133 Italy

## Abstract

Although it is becoming increasingly evident that maternal starvation during pregnancy can have permanent effects on a range of physiological processes in the offspring, scant information is available about the consequence of such condition for oogenesis and hence for lifetime reproductive success of progeny in mammals. In the present study, we address this topic by starving pregnant mice at the time of ovarian differentiation (12.5 days post coitum (dpc)) for three consecutive days and analyzed the consequence first on the survival of the fetal oocytes and their capability to progress throughout the stages of meiotic prophase I (MPI) and then on the postnatal folliculogenesis of the offspring. The results showed that maternal starvation increased apoptosis in the fetal ovaries, resulting in reduction of the oocyte number. Moreover, MPI progression was slowed down in the surviving oocytes and the expression of DNA repair players in the starved ovaries increased. Transcriptome analysis identified 61 differentially expressed genes between control and starved ovaries, the most part of these being involved in metabolic processes. A significant decrease in the percentage of oocytes enclosed in primordial follicles and the expression of oocyte genes critically involved in folliculogenesis such as *Nobox, Lhx8* and *Sohlh2* in the 3 days post partum (dpp) starved ovaries were found. Finally, at the time of juvenile period (21 dpp), the number of oocytes and antral follicles resulted significantly lower in the ovaries of the offspring from starved mothers in comparison to controls. Our findings support the notion that maternal starvation can affect ovary development in the offspring that could adversely affect their reproductive success in the adult life.

## Introduction

Adequate and correct diet during pregnancy are critical for the health of mother and newborns^1–3^. As a matter of fact, it had been clearly established that some offspring pathologies (for instance, obesity, diabetes and cardiovascular disease) might have their origins in inadequate nutrition during pregnancy^4,5^. However, little information is available about the effect of starvation during pregnancy on the offspring reproductive functions in mammals.

In humans, three studies examined whether exposure to acute, severe famine in utero during the Dutch famine 1944–1945 affected a women’s subsequent reproduction. Lumey and Stein^6^ found that exposure to fetal famine was sufficient to result in a 300 g decrease in mean birthweight; however, these individuals did not suffer from adverse effects on their subsequent fecundity in adulthood, but were more likely to give birth to offspring of reduced birthweight. This reduced birthweight in the second generation was associated with a high frequency of early infant mortality. Painter et al.^7^, who interviewed the same sample of women at a mean age of 50 years, but used a different sample of controls, found a small but significant decrease in the prevalence of nulliparity. More recently, Yarde et al.^8^, in an independent sample of women born after the same famine, reported earlier menopause. Elias et al.^9^ found a slight decrease in age of menopause following famine exposure during early childhood. In a follow-up study of women born in England in the first half of the twentieth century, Cresswell et al.^10^ and Hardy and Kuh^11^ also found that menstruation ceased at an earlier age in those who had low weight gain during their first or the second year of life (but see Treloar et al.^12^, for the opposite result). Steiner et al.^13^ reported a weak association between birthweight and age at menopause. Some authors observed that low birthweight infants with prematurity or growth retardation tend to have fewer offspring^14,15^, and that retarded fetus growth can impair ovarian development, which may have implications for the timing of menopause^16^. Despite such apparent contradictory results in humans, animal models support the notion that starvation during pregnancy can have adverse effect on the offspring reproductive capability. A reduction of lifetime reproductive capacity after prenatal undernutrition has been reported in female mice^17^ and sheep^18^. Food restriction during the second half of pregnancy in rats resulted in premature reproductive senescence in female offspring^19^. In single-ovulating species, a study found evidence that maternal dietary restriction influences ovarian reserve in bovine^20^. Because female reproductive capacity in rodents is largely defined by the number and quality of primordial follicles developed in the ovary during the neonatal period, termed the ovarian reserve, it can be hypothesized that reduced amount of nutrients during this crucial process can impair the formation of the ovarian reserve with adverse consequence for reproduction. Actually, several studies showed that apoptosis and autophagy are part of the starvation cell response, possibly triggered by oxidative stress in damaged cells^21^ and that insulin-like growth factor-1 signaling plays an important role in such processes^22^. In this regard, we recently found that starvation at birth impairs germ cell cyst breakdown and increases autophagy and apoptosis in mouse oocytes^21^. Interestingly, diet can also cause epigenetic changes in gene expression with possible consequence for the correct development of cells and tissues^23^. Epigenetics involves heritable changes in gene expression via post-translational and post-transcriptional modifications without altering DNA base sequence. For instance, in the mouse, gestational nutritional restriction has been reported to alter the level of DNA methylation in the sperm of male offspring, resulting in metabolic diseases in the next generations^23–25^.

In the present study, pregnant mice at the time of the gonad sex differentiation (12.5 days post coitum (dpc)) experienced a starvation period of 3 days, and the consequences on the ovary development during the fetal period and early folliculogenesis were investigated.

## Results and discussion

Many animals alter their reproductive strategies in response to environmental stress. For example, in female *Drosophila and Caenorhabditis elegans*, starvation activates apoptotic checkpoints and autophagy in oogenesis and reduces the production of mature oocytes^26,27^. In this regard, we recently found that nutrient deficiency at birth could generate a number of adaptive metabolic and oxidative responses in the ovaries causing increased apoptosis and autophagy in both the somatic cells and oocytes, leading to a delay of germ cell cyst breakdown and follicle assembly^21,28^. Here we investigated the consequences of starvation of pregnant mice for 3 days at the time of the fetal gonad sex differentiation and beginning of meiosis on the ovary development during the fetal period and early folliculogenesis.

### Mother’s starvation impairs fetal growth

**Fig. 1:**
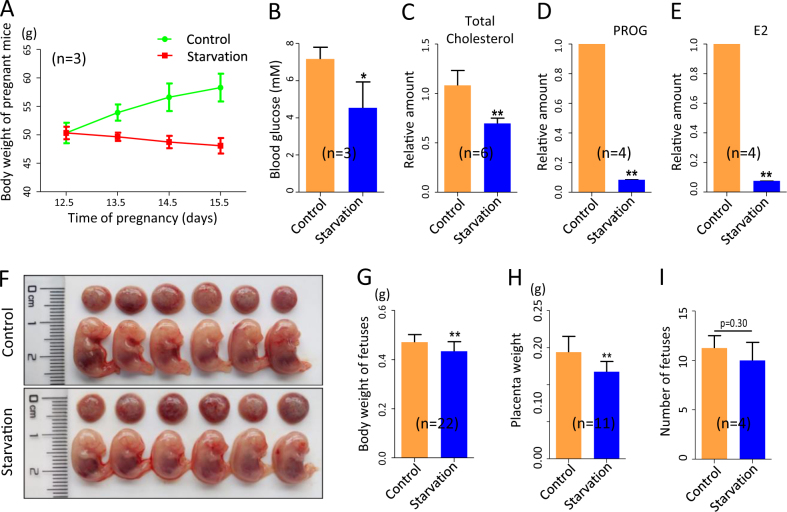
Effects of starvation on mothers and fetuses. **a** Body weight of pregnant females from 12.5 to 15.5 dpc in control and starved groups. **b**–**e** Amount of glucose, total cholesterol, PROG and E2 in the blood of 15.5 dpc pregnant females in control and starved groups. **f** The 15.5 dpc fetuses and placentas. **g**,** h** Body and placenta weights of 15.5 dpc fetuses. **i** Number of fetus of 15.5 dpc pregnant females; *n* = number of mothers or fetuses (**g**,** h**) used for the analyses. Results are presented as mean ± SEM. *P < 0.05; **P < 0.01

Starvation for a relative short pregnancy time, from 12.5 to 15.5 dpc, caused a significant decrease of the mother’s body weight at the end of treatment (Fig. 1a), paralleled to reduced concentrations of glucose, total cholesterol, progesterone (PROG) and E2 in the blood (Fig. 1b–e). The greatly reduced level of PROG was likely responsible for the slightly higher probability of pregnant female abortion at 15.5 dpc (data not shown). Moreover, the body weight of the surviving fetuses was significantly lower in the starved (0.43 ± 0.008 g) compared to control (0.47 ± 0.007 g) groups (Fig. 1f, g, *P* < 0.01). Finally, the weight of placenta also resulted significantly lower in the starved (0.12 ± 0.004 g) than in the control (0.14 ± 0.006 g) groups (Fig. 1h). For non-abortion mice after starvation, however, there was no significant difference in the number of the delivered pups compared with that of control group (11.25 ± 0.63 vs 10.00 ± 0.91) (Fig. 1i).

### Mother’s starvation increases apoptosis in the fetal ovaries and causes reduction of the oocyte number

**Fig. 2:**
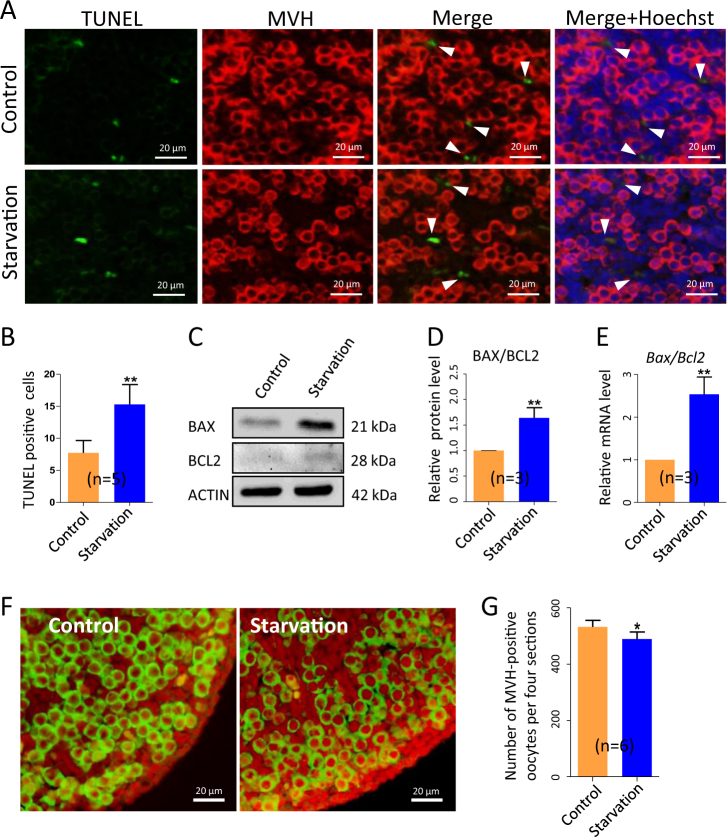
Apoptotic markers increase and oocyte numbers decrease in 15.5 dpc ovaries from fetuses of starved mothers. **a** TUNEL immunohistochemistry (green) in MVH-positive oocytes (red); cell nuclei was stained with Hoechst (blue). **b** Number of TUNEL-positive cells. **c**–**e** WB and qRT-PCR for BAX and BCL2. **f**, **g** MVH immunofluorescence (green) and number of oocytes in control and starved ovaries; *n* = number of ovaries (**g**) or independent repeats used for the analyses. *P < 0.05; **P < 0.01

In line with our previous results reported above^21^ and general observation that starvation, as a result of metabolic stress, increases autophagy and/ or apoptosis in several tissues^29^, we found evidence of increased level of apoptosis in the ovaries of the starved 15.5 dpc fetuses. In fact, the number of TUNEL (terminal deoxynucleotidyl transferase dUTP nick end labeling)-positive cells scored per unit area in tissue sections of ovaries of the starved fetuses was almost double (15.30 ± 1.39) in comparison to control (7.73 ± 0.86) (Fig. 2a, b). At the same time, the *Bax/Bcl-2* ratio, evaluated at both the messenger RNA (mRNA) and protein levels, resulted significantly higher apoptosis in the ovaries of the starved fetuses in comparison to control (Fig. 2c–e).

The increased levels of apoptosis markers in the starved ovaries were associated to a slight but statistically significant decreased number of oocytes assessed in tissue sections after staining with the germ cell-specific anti-mouse VASA homolog (MVH) antibody (489.30 ± 10.16 vs 532.40 ± 9.48, *P* < 0.05) (Fig. 2f, g). It remained to determine if such reduced oocyte number was due to increased apoptotic rate in oocytes or an indirect consequence of increased apoptosis in the surrounding somatic cells.

### Mother’s starvation delays meiotic progression in fetal oocytes and increases the expression of DNA repair players in the starved ovaries

**Fig. 3:**
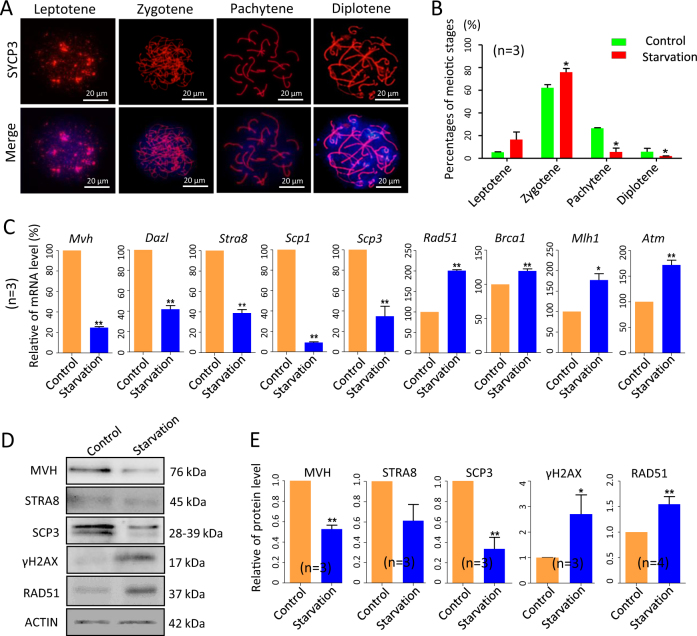
Slowed MPI progression and increased DNA repair players in 15.5 dpc ovaries from fetuses of starved mothers. **a** SCP3 immunofluorescence (red) of oocyte cytospread at various MPI stages; nuclei was counterstained with Hoechst (blue). **b** Percentages of oocyte at different MPI of 15.5 dpc ovaries. **c**–**e** qRT-PCR and WB analyses of the expressions of germ cell-specific, meiotic- and DNA damage-related genes in control and starved ovaries; *n* = number of independent repeats used for the analyses. *P < 0.05; **P < 0.01

In the fetal ovaries of the CD-1 mice, between 12.5 and 14.5 dpc, most part of germ cells enter meiosis and begin to progress through the meiosis prophase I (MPI) stages^30^. To evaluate if starvation affects these processes, we analyzed MPI stages by oocyte cytospreads. The results showed that MPI progression was altered in starved (oocytes at zygotene 75.92 ± 3.25%; oocytes at pachytene: 5.61 ± 3.41%) compared with control ovaries (oocytes at zygotene: 62.21 ± 2.73%; oocytes at pachytene: 26.56 ± 0.37%) (Fig. 3a, b). Moreover, quantitative real-time PCR (qRT-PCR) analyses showed that the transcripts for the germ cell-specific genes, *Mvh* and deleted in azoospermia-like (*Dazl*), and of the meiosis-related genes, stimulated by retinoic acid 8 (*Stra8*), synaptonemal complex protein 1 and 3 (*Scp1* and *Scp3*), were all significantly decreased in the starved ovaries (Fig. 3c). These results were also confirmed at the protein level for MVH, STRA8 and SCP3 (Fig. 3d, e). Reduction of the oocyte number and delay in MPI progression in the starved ovaries could both explain such decreased gene expressions. The absence of significant difference in the percentages of oocytes at different MPI stages in the ovary of control and treated groups at 18.5 dpc (see below), suggested, however, a recovery in meiotic progression after the mother resuming the correct diet in the last period of pregnancy.

Interestingly, western blot (WB) analyses showed significant higher levels of γH2AX (a marker of DNA double-strand breaks) and RAD51 (involved in DNA double-strand breaks repair), in the starved 15.5 dpc ovaries (Fig. 3d, e). Moreover, besides *Rad51*, we found that starvation increased the transcript levels of other genes involved in DNA repair and homologous recombination such as *Atm* (*ataxia telangiectasia mutated*), *Mlh1* (*mutL homolog 1*) and *Brca1* (*breast cancer type 1 susceptibility protein*) (Fig. 3c).

**Fig. 4:**
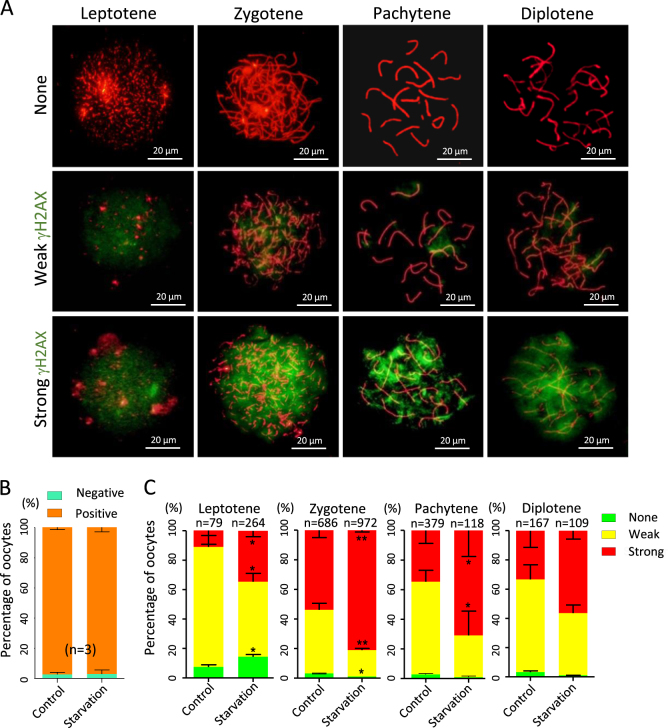
γH2AX staining in oocytes at various MPI stages. **a** Representative SCP3 (red) and γH2AX (green) staining of oocyte cytospread at various MPI stages. **b**, **c** Quantification of γH2AX-positive oocytes in the ovaries of fetuses from control and starved mothers; *n* = number of independent repeats or counting oocytes (**c**) used for the analyses. *P < 0.05; **P < 0.01

**Fig. 5:**
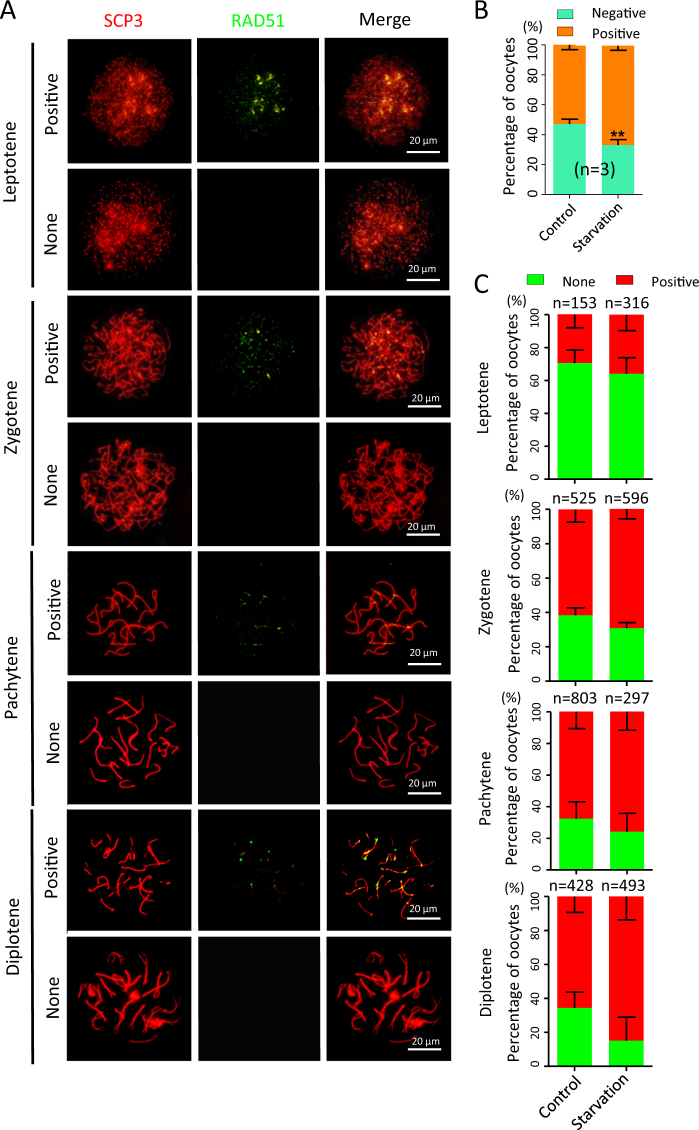
RAD51 staining in oocytes at various MPI stages. **a** Representative SCP3 (red) and RAD51 (green) staining of oocyte cytospread at various MPI stages. **b**,** c** Quantification of RAD51-positive oocytes in the ovaries of fetuses from control and starved mothers; *n* = number of independent repeats or counting oocytes (**c**) used for the analyses. **P < 0.01

Double staining of oocyte cytospreads for SCP3 and γH2AX (Fig. 4a) showed that while there was no significant difference in the total percentage of γH2AX-positive oocytes between the control (97.34 ± 0.80%) and the starved (97.11 ± 1.60%) groups (Fig. 4b), in the latter, at all MPI stages analyzed, the percentages of oocytes showing strong γH2AX was much higher at varying degrees except diplotene stage (Fig. 4c). Finally, double staining for SCP3 and RAD51 revealed that RAD51-positive rate significantly increased (*P* < 0.01; Fig. 5a, b), but there were not significant differences in the number of positive oocytes between the control and starved groups at each MPI stage (Fig. 5c).

These last results evidence that defects in MPI likely occur because of alteration of homologous recombination and DNA repair in the oocytes of the starved ovaries. This supports the important notion that nutrients might modulate cellular DNA damage response. The relevance of diet and nutrition in human health and disease is well established. Actually, basic laboratory researches, clinical trials and epidemiological studies demonstrated that nutrient-rich bioactive foods can induce epigenetic changes and alter gene expression by the alteration of the histone structure, DNA methylation, miRNA modulation and DNA repair^31,32^.

### Transcriptome analysis and comparison identify differentially expressed genes between control and starved ovaries

In transcriptome analyses, we found that control and starved ovaries showed 61 significant differentially expressed genes (DEGs) (Fig. S1A-S1C) and that most part of these genes were involved in metabolic process (Fig. S1D).

**Fig. 6:**
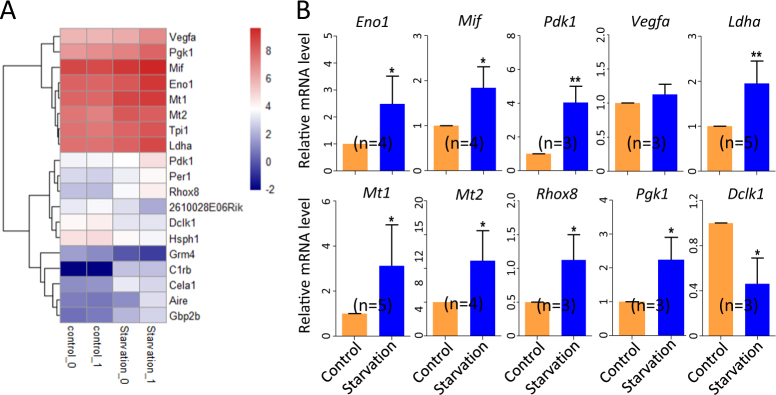
Transcriptome analysis of 15.5 dpc ovaries from fetuses of control and starved mothers. **a** Heatmap of differentially expressed genes (DEGs) constantly affected by maternal starvation. **b** qRT-PCR analyses of 10 genes showed in the heatmap in 15.5 dpc control and starved ovaries; *n* = number of independent repeats used for the analyses. *P < 0.05; **P < 0.01

For DEGs that resulted more constantly affected by maternal starvation, a heatmap was generated (Fig. 6a). qRT-PCR was then performed for 10 of these genes potentially relevant for reproductive processes (Fig. 6b). The transcripts of most of these genes, such those for *enolase 1* (*Eno1*)^33^, *macrophage migration inhibitory factor* (*Mif*)^34^, *pyruvate dehydrogenase kinase 1* (*Pdk1*)^35^, *lactate dehydrogenase A* (*Ldha*)^36^, melatonin receptors *Mt1* and *Mt2*^37^, *reproductive homeobox 8* (*Rhox8*)^38^ and *phosphoglycerate kinase 1* (*Pgk1*)^39^, were significantly increased. Conversely, mRNA amount of *doublecortin-like kinase 1* (*Dclk1*)^40^ was decreased while those of *vascular endothelial growth factor A* (*Vegfa*)^41^ was not affected.

### Mother’s starvation causes reduction in the number of the primordial follicles and impairs follicle development in the offspring ovaries

To investigate whether the defects in the oogenic process found in the starved 15.5 dpc fetal ovaries impaired the ovary development at later stages, we examined MPI in oocytes obtained from 18.5 dpc starved ovaries, and folliculogenesis in 3 and 21 days post partum (dpp) ovaries of offspring of starved mothers.

**Fig. 7:**
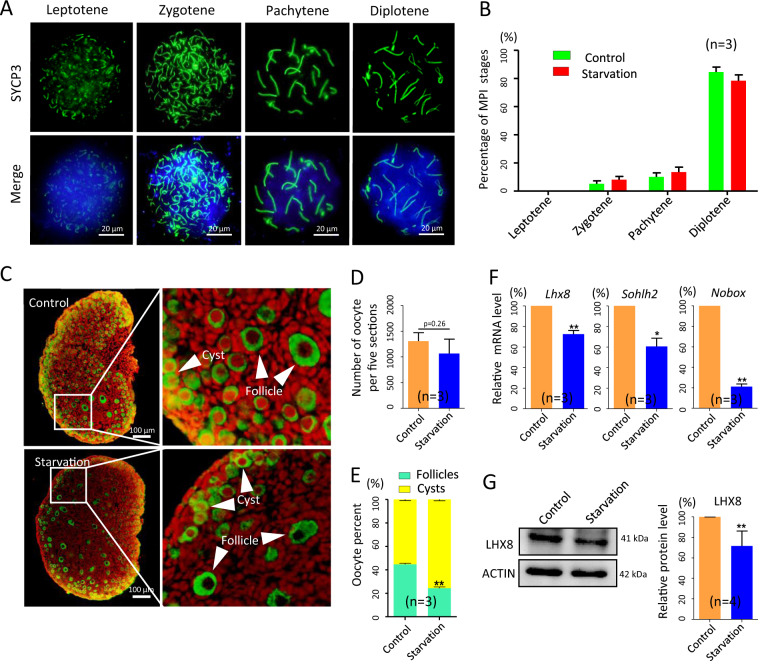
MPI oocyte stages and folliculogenesis in the ovaries of 18.5 dpc fetuses and 3 dpp pups from control and starved mothers. **a** Representative oocyte cytospreads stained for SCP3 (green); nuclei counterstained with Hoechst (blue). **b** Percentages of oocyte in MPI stages. **c** Sections of 3 dpp ovaries showing MVH-positive oocytes (green) in cysts (white arrows) or in primordial follicles (white arrowheads). **d**,** e** Number of oocytes and percentages of oocyte in cysts or in primordial follicles in 3 dpp ovaries. **f**,** g** qRT-PCR and WB of oocyte-specific genes involved in folliculogenesis; *n* = number of ovaries (**d**,** e**) or independent repeats used for the analyses. *P < 0.05; **P < 0.01

While, as reported above, at 18.5 dpc, no significant difference in the oocyte MPI stages between control and starved ovaries was found (Fig. 7a, b), in the latter, at 3 dpp, we scored a slight decrease in the oocyte number (Fig. 7c, d), but above all a marked lower percentage of oocytes enclosed in primordial follicles in comparison to the control (24.31 ± 1.15% vs 44.65 ± 1.01%, *P* < 0.01; Fig. 7e). The parallel decreased amount of mRNA of oocyte genes involved in the germ cell cyst breakdown and primordial follicle formation such as *Nobox* (*newborn ovary homeobox*)*, Sohlh2* (*spermatogenesis and oogenesis specific basic helix-loop-helix*2) (Fig. 7f) and *Lhx8* (*LIM homeobox 8*, protein level in Fig. 7g) in the 3 dpp starved ovaries offered a likely explanation of this defect (*P* < 0.05 or *P* < 0.01).

**Fig. 8:**
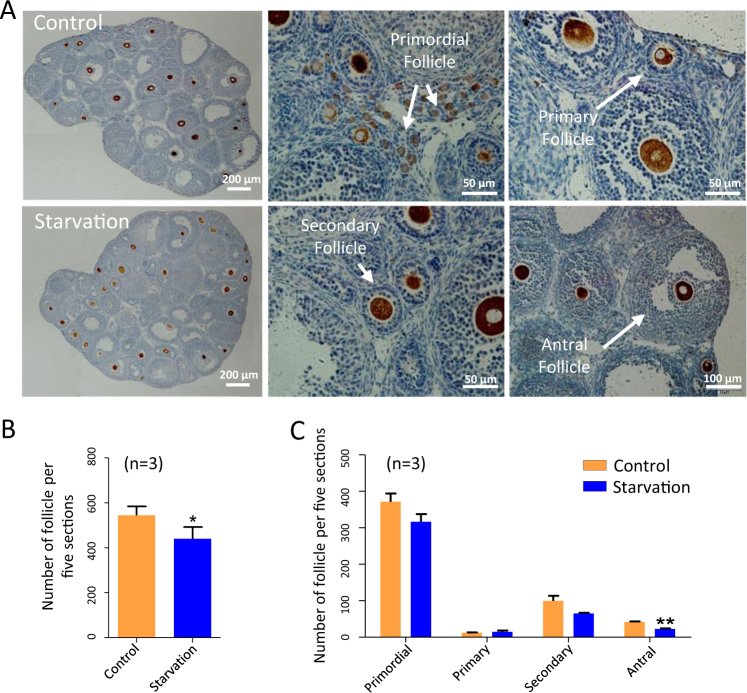
Analysis of folliculogenesis in 21 dpp ovaries of offspring of starved pregnant mothers. **a** Representative immunohistochemistry of MVH-positive oocytes (dark brown) in 21 dpp ovaries. **b**,** c** Quantification of the number of oocytes and different classes of follicles in 21 dpp ovaries of offspring of control and starved mothers; *n* = number of ovaries used for the analyses. *P < 0.05; **P < 0.01

Finally, at 21 dpp, the number of follicles in tissue sections resulted significantly lower in starved ovaries in comparison to control (440.5 ± 25.94 vs 544.6 ± 17.67) (*P* < 0.05; Fig. 8a, b), while the number of antral follicles showed a significant decrease of almost one half in the ovaries of the offspring from starved mothers (22.67 ± 1.67 vs 42.00 ± 1.53) (*P* < 0.01; Fig. 8c). Although these last observations are predictive of reduced fertility performance, at the moment no detailed information is available about such possible defect.

**Fig. 9:**
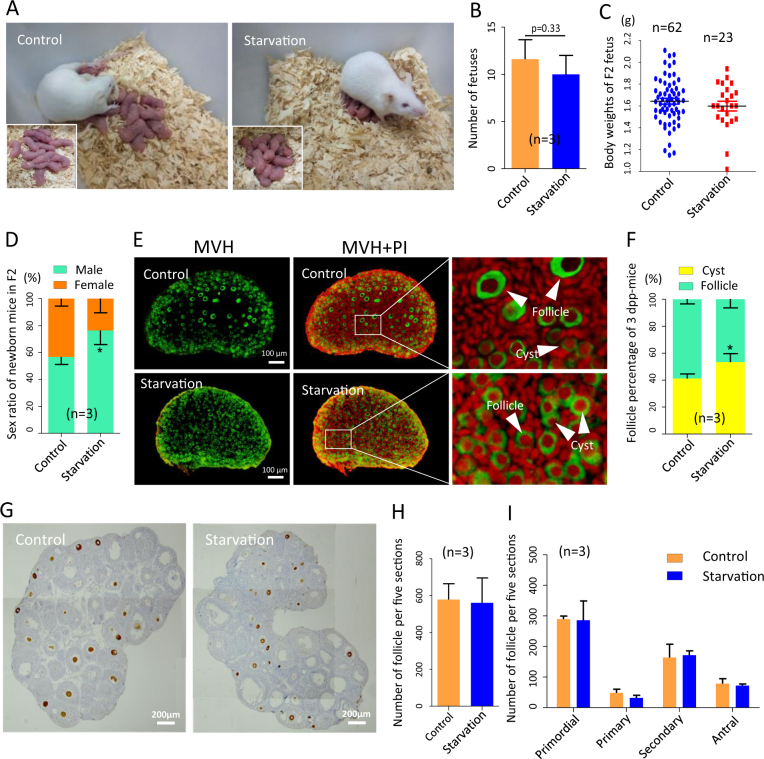
Analysis of folliculogenesis F2 offspring of starved pregnant mothers. **a** Photograph of F2 offspring of control and starved mothers. **b** Litter size of F1 mothers. **c** Body weight of F2 fetus at 0 dpp. **d** Sex ratio of F2 offspring. **e**, **f** MVH-positive oocytes (green) in cysts (white arrows) or in primordial follicles (white arrows) in 3 dpp ovaries. **g**–**i** Quantification of the number of follicles and percentages of different classes of follicles in 21 dpp ovaries from control and F2 offspring of starved ovaries; *n* = number of ovaries or fetuses (**c**) or independent repeats (**b**, **d**) used for the analyses. *P < 0.05

On the other hand, the litter size and birthweight of F2 offspring produced from F1 females scarcely declined (Fig. 9a–c), while there was an apparent imbalance in the sex ratio at birth (*P* < 0.05; Fig. 9d). Moreover, the percentages of oocyte enclosed in cysts and follicles suggested a small depletion in the formation of primordial follicles at 3 dpp (*P* < 0.05; Fig. 9e, f), whereas the total number of follicles and progression of follicular growth between starved and control groups had no significant defects at 21 dpp (Fig. 9g–i).

In conclusion, our study represents a first indication that even a relatively short period of mother starvation during critical stages of the ovary development in the fetuses can disturb oogenic processes with adverse consequence for folliculogenesis.

## Materials and methods

### Animal breeding and treatment

All animals used in this study were CD-1 mice, which were kept in self-help feeding way with light and dark cycle of half day and raised according to the national guideline and Ethical Committee of Qingdao Agricultural University. Mating was arranged regularly at 4:30–5:30 p.m., vaginal plug was checked at 8:30–9:30 a.m. on next morning; the presence of vaginal plug was considered as 0.5 dpc.

Due to the sensibility to food deprivation between 12.5 and 15.5 dpc, which is crucial for the ovarian sex differentiation and meiosis initiation, starvation began at 11:00–12:00 a.m. at 12.5 dpc and ceased at the same time at 15.5 dpc. During this process of starvation, the water was provided as usual and food supply was cut down, and after that the diet of starved females came back to normal. The total number of starved mothers was 120. These mothers, unless otherwise indicated, were killed to remove the fetuses.

### Detection of blood sugar, total cholesterol and sex hormone levels

Biochemical indicators designed to validate the effect of starvation on the pregnant mothers were: blood sugar, total cholesterol, PROG and 17β-estradiol (E2) levels. The blood sugar concentration was determined by collecting blood samples with the ACCU-CHEK^®^ Active of Roche (Roche, Germany) and corresponding test strips. The levels of total cholesterol (Comin Biotechnology, Suzhou, China), PROG (LanpaiBIO, shanghai, China) and E2 (Jinma Biotechnology, Shanghai, China) were tested following the manufacturer’s instructions.

### Quantitative real-time PCR

The complementary DNA (cDNA) prepared for PCR was synthesized by TransScript One-Step gDNA Removal and cDNA Synthesis SuperMix (TransGen, AT311-03, Beijing, China) from total extracted RNA using RNAprep pure Micro Kit (Aidlab, RN07, Beijing, China). All primers used for analysis were listed in Table S1. PCR amplification was conducted with SYBR^®^ Premix Ex Taq™ II (TAKARA, RR820A, Japan) and Light Cycler real-time PCR instrument of Roche 480 (Roche, Germany). Each sample extracted from 3 to 6 ovaries was amplified in triplicate to normalize the system and pipetting error, and the relative mRNA expression levels of all genes were calculated by the formula of 2^−ΔΔCt^ and normalized with *β-actin*.

### Analysis of meiotic stages

The meiotic stages of fetal oocytes were analyzed using cytospreads as previously described^42,43^. Fixed samples were blocked with antibody dilution buffer (ADB) at 37 °C for 30 min and incubated with primary antibodies (Table S2) at 37 °C for 8 h in a 1:200 dilution. After three washes with Tris-buffered saline (TBS), they were blocked at 4 °C overnight in ADB. Finally, the cells were incubated in dark with secondary antibodies of CY3 (anti-rabbit, Beyotime, A0562, Nantong, China; anti-mouse, Beyotime, A0568) or fluorescein isothiocyanate (FITC)-labeled goat IgG (anti-rabbit, Beyotime, A0516; anti-mouse, Beyotime, A0521) at 37 °C for 1.5 h and stained with Hoechst 33342 (Beyotime, C1022) for 5 min. Slides were sealed and prepared for fluorescence microscope observation (Olympus, BX51, Japan). The number of ovaries of each sample was 3 to 4 and every independent experiment for oocyte counting was at least 300.

### Immunohistochemistry and TUNEL assay

Ovaries were fixed in 4% paraformaldehyde (Solaibio, P1110, Beijing, China) and processed according to standard methods for paraffin samples. Sections were taken every 5 µm and immunohistochemistry performed on deparaffinated sections after antigen retrieval at 96 °C. Sections were blocked and incubated with anti-MVH protein antibody as reported above for primary antibodies, then FITC (CY3 in TUNEL assay)-labeled goat anti-rabbit IgG and nucleic acid was stained with propidium iodide (Solaibio, P8080-10, Beijing, China) or Hoechst 33342 (in TUNEL assay). For immunohistochemistry with enzyme substrates, the samples must be treated with 3% H_2_O_2_ before primary antibody incubation, and then the secondary antibody was horse radish peroxidase (HRP)-conjugated goat anti-rabbit IgG (Beyotime, A0258); the DAB kit was purchased from ZSGB-BIO company (Beijing, China). Considering that the oocyte number fluctuated largely in ovaries of different size, the oocyte of each experimental ovary was counted in every five sections and at total three sections from different fetuses or six for pups. Then, Image J software was used to calculate areas of these ovary sections, and accordingly the oocyte counting was adjusted to the same level on the basis of ovarian section areas.

For the estimate of the number of the different classes of follicles in postnatal ovaries, we defined the primordial and primary follicle of a follicle containing an intact MVH-positive oocyte surrounded by a single layer of flat or cuboidal granulosa cells, the secondary and mature follicles were two or multiple layers of granulosa cells, and the antral follicle was a kind of follicle with a fluid-filled cavity adjacent to the oocyte^44^: the total number of each follicle class was counted in every five sections for a total of 8 or 10 sections for each of three ovaries from different mice.

The TUNEL assay was performed on paraffin ovary sections as before^43^, using the BrightGreen Apoptosis Detection Kit (Vazyme, A112-02, Nanjing, China) according to the manufactures’ instructions.

### Western blot

For each sample, proteins were extracted from 3 to 4 ovaries using the RIPA lysis solution (Beyotime, P0013C) for sodium dodecyl sulfate–polyacrylamide gel electrophoresis. They were then transferred onto a polyvinylidene fluoride membrane (immobilon-P^SQ^ transfer membranes, Millipore, ISEQ00010, USA). After blocking in TBST buffer (TBS with Tween-20) containing 5% bovine serum albumin, the membrane was incubated in primary antibody (Table S2) overnight at 4 °C, washed and incubated with HRP-conjugated goat anti-rabbit (Beyotime, A0208) or anti-mouse IgG (Beyotime, A0216) diluted in TBST at room temperature for 1.5 h. Ultimately, the BeyoECL Plus Kit (Beyotime, A0018) was used for chemiluminescence; β-ACTIN was used as housekeeping protein as previous reported^21^.

### Transcription data analysis

A total of 10–12 ovaries were collected from fetuses, subjected to total RNA extraction and sequenced. The latter was obtained from ANOROAD company with Illumina Hiseq 2000 sequencing system (San Diego, CA, USA). The bioinformatic analysis was carried out as previously described^45–47^. With the transcriptome data assembly with Cufflinks and calculation of gene expression levels with Cuffdiff, the differentially expressed mRNAs were defined as the expression levels of mRNAs with *q*-value < 0.05, and the data visualization was conducted with R Bioconductor/CummeRbund packages^48^. In addition, the biofunction of these differentially expressed genes was analyzed via Gene Ontology (GO) enrichment based on GO Database^49^.

### Statistical analysis

Results obtained from at least three independent experiments were expressed as mean ± SEM. Data were statistically analyzed with Graph Pad Prism software and the significant difference was determined with Student’s unpaired *t*-test of independent sample. *P* < 0.05 was considered as significantly different, while *P* < 0.01 was a highly significant difference.

## Electronic supplementary material

Supplementary figure

Supplementary figure legends

Supplementary tables
