# Supplementary figures and images for: Starvation during pregnancy impairs fetal oogenesis and folliculogenesis in offspring in the mouse
Source: Cell Death Dis. 2018 Apr 18;9(5):452. doi: 10.1038/s41419-018-0492-2 (PMC5906686; doi:10.1038/s41419-018-0492-2)

Supplemental Figure S1

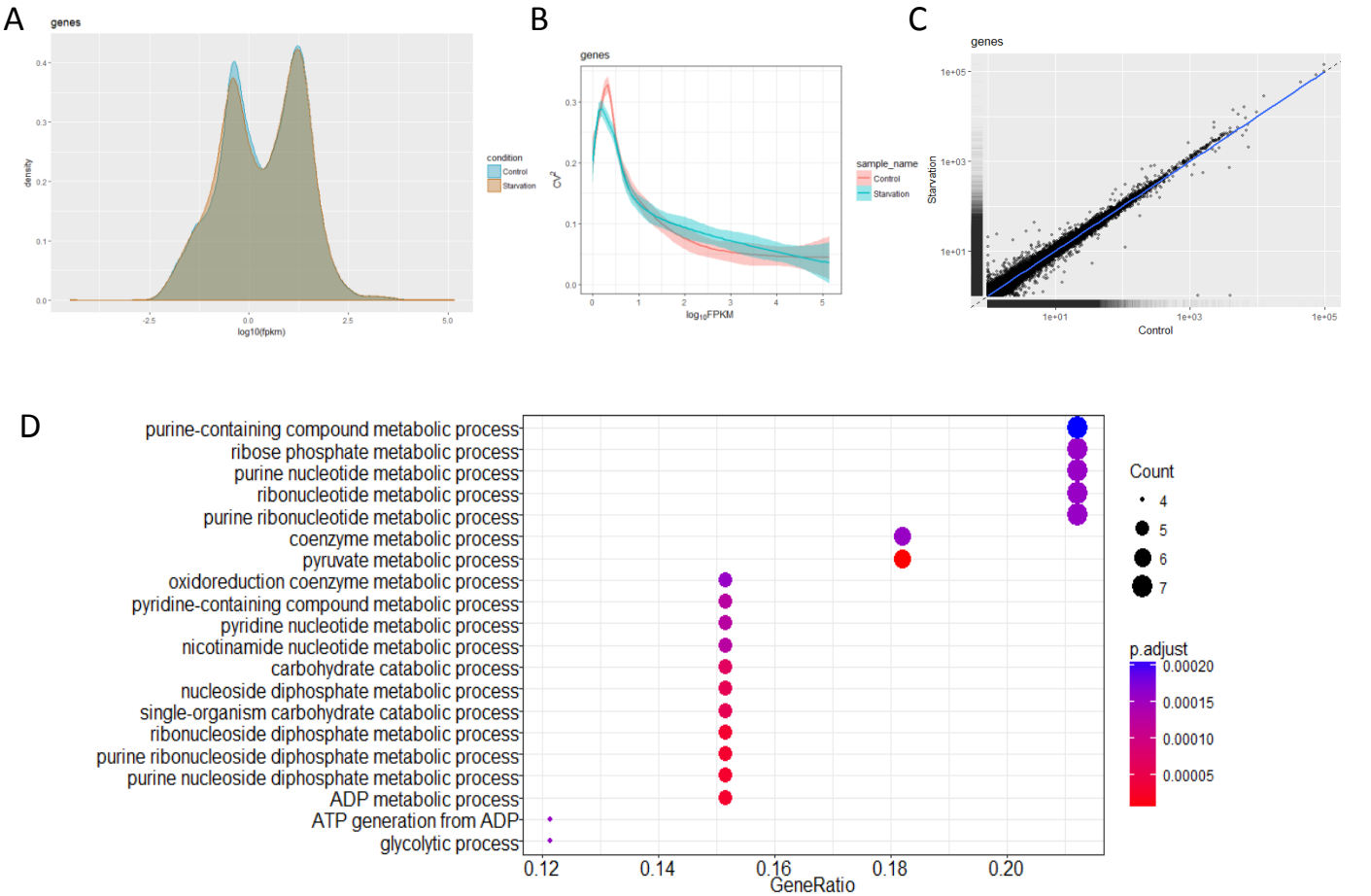

Supplement: Supplementary file 1 — Supplementary figure [file 41419_2018_492_MOESM1_ESM.pdf]
