# Supplementary material for: Starvation during pregnancy impairs fetal oogenesis and folliculogenesis in offspring in the mouse
Source: Cell Death Dis. 2018 Apr 18;9(5):452. doi: 10.1038/s41419-018-0492-2 (PMC5906686; doi:10.1038/s41419-018-0492-2)
Supplement: Supplementary file 2 — Supplementary figure legends [file 41419_2018_492_MOESM2_ESM.docx]

**Supplemental Figure**

**Figure S1.** **Transcriptome analysis of 15.5 dpc control and starved ovaries.**

A) Density plot of transcriptome sequencing data in 15.5 dpc control and starved ovaries; B) Squared coefficient of variation in control and starved groups; C) Pairwise scatterplots in gene expression of control and starved groups; D) Gene ontology (GO) analysis of 61 DEGs in control and starved groups.
