# Supplementary material for: Starvation during pregnancy impairs fetal oogenesis and folliculogenesis in offspring in the mouse
Source: Cell Death Dis. 2018 Apr 18;9(5):452. doi: 10.1038/s41419-018-0492-2 (PMC5906686; doi:10.1038/s41419-018-0492-2)
Supplement: Supplementary file 3 — Supplementary tables [file 41419_2018_492_MOESM3_ESM.docx]

**Supplementary Tables**

**Table S1. Primers Used for Quantitative RT-PCR**

| **Genes** | **Genbank** | **Forward primer sequence** | **Reverse primer sequence** | **Product Length (bp)** |
| --- | --- | --- | --- | --- |
| *Eno1* | NM_023119.2 | AGATCTTTGACTCCCGTGG | GAGACACCCTTCCCCATGAA | 160 |
| *Mif* | NM_010798.2 | GCTCATGACTTTTAGCGGCA | GGCAGCGTTCATGTCGTAAT | 178 |
| *Pdk1* | NM_008828.3 | GATGCTTTTGGGACTGCACA | TCAGCTGGATCTTGTCTGCA | 181 |
| *Vefga* | NM_001287058.1 | CACGACAGAAGGAGAGCAGA | GGGCTTCATCGTTACAGCAG | 188 |
| *Ldha* | NM_010699.2 | TGGCTTGGAAAATCAGTGGC | AAGAGACTTCAGGGAGACGC | 206 |
| *Mt1* | NM_013602.3 | GGACCCCAACTGCTCCTG | CTTTGCAGACACAGCCCTG | 152 |
| *Mt2* | NM_008630.2 | CTGTGCCTCCGATGGATCC | CTTGTCGGAAGCCTCTTTGC | 151 |
| *Rhox8* | NM_001004193.2 | GCCGCCAGAGATGAAACTAC | CCTGGAGCTGGAACTTGGTA | 202 |
| *Pgk1* | NM_008828.3 | GATGCTTTTGGGACTGCACA | TCAGCTGGATCTTGTCTGCA | 181 |
| *Dclk1* | NM_001195540.1 | CAGCTCTCTACTCCACGCTC | AAATCATCCGACGAGAGGGG | 107 |
| *Nobox* | NM_130869.3 | CTATCCTGACAGTGACAAACGCC | CACCCTCTCAGCACCCTCATTAT | 251 |
| *Lhx8* | NC_000069.5 | CAGTTCGCTCAGGACAACAA | CCTGCAGTTCTGAAACCACA | 105 |
| *Sohlh2* | NM_028937.3 | TCTCAGCCACATCACAGAGG | GGGGACGCGAGTCTTATACA | 199 |
| *Bax* | NM_007527 | ATGCGTCCAAGGAAGACTGAG | CCCCAGTTGAAGTTGCCATCAG | 162 |
| *Bcl-2* | NM_009741 | GCAGAGATGTCCAGTCAG | CACCGAACTCAAAGAAGG | 127 |
| *Actin* | NM_007393.3 | TCGTGGGCCGCTCTAGGCAC | TGGCCTTAGGGTTCAGGGGGG | 255 |
| *Mvh* | NM_001145885.1 | AGGGGATGAAAGAACTATGGTC | AGCAACAAGAACTGGGCACT | 175 |
| *Dazl* | NM_010021.5 | ATCAGCAACCACAAGTCAAGG | GAGACAAATCCATAGCCCTTCG | 192 |
| *Stra8* | NM_009292.1 | CTCCTCCTCCACTCTGTTGC | GCGGCAGAGACAATAGGAAG | 135 |
| *Scp1* | NM_011516.2 | GCGAAGATTGCTTTGGAGAC | GCAGATGCCCGCAGATTAT | 296 |
| *Scp3* | NM_011517.2 | GGGGCCGGACTGTATTTACT | AGGCTGATCAACCAAAGGTG | 169 |
| *Rad51* | NM_011234.4 | ACCAGACCCAGCTCCTTTAC | CAAGTCGAAGCAGCATCCTC | 171 |
| *Brca1* | NM_009764.3 | ATCCCGGGAAAAGCTCTTCA | GGCTGCACGATCACAACTAG | 171 |
| *Mlh1* | NM_026810.2 | TTGCCAACCTGCCAGATCTA | ATTTGCAGCCAATCCACAGG | 231 |
| *Atm* | NM_007499 | TCGATCTCATGAAGCCCCTC | CAATCCGTGTGCTCTCCATG | 234 |
| *Spoll* | NM_001305434.1 | TACTGCTGTGCCGACTAACA | GTAGGGATCTGCATCGACCA | 232 |

**Table S2. Primary Antibodies**

| **Antibody** | **Vendor; Cat. No.** | **Dilution** | **Applications** |
| --- | --- | --- | --- |
| MVH (VASA) | Abcam; ab13840 | 1:200 | Immunohistochemistry |
|  |  | 1:1000 | Western Blot |
| BAX | Cell signaling; #2772S | 1:1000 | Western Blot |
| BCL-2 | Beyotime; AB112 | 1:1000 | Western Blot |
| SCP3(Rb) | Novus Biologicals; NB300-232 | 1:1000 | Western Blot |
|  |  | 1:200 | Immunofluorescence |
| SCP3(Mus) | Abcam; ab97672 | 1:200 | Immunofluorescence |
| STRA8 | Abcam; ab49602 | 1:1000 | Western Blot |
| γH2AX | Abcam, ab26350 | 1:1000 | Western Blot |
|  |  | 1:200 | Immunofluorescence |
| RAD51 | Abcam; ab133534 | 1:1000 | Western Blot |
|  |  | 1:200 | Immunofluorescence |
| LHX8 | Sigma; SAB2101342 | 1:1000 | Western Blot |
| β-ACTIN | Sangon Biotech; D110001 | 1:1000 | Western Blot |
